# Prevalence and Indications of Cesarean Section in a Community Hospital of Western Region of Nepal

**DOI:** 10.31729/jnma.3760

**Published:** 2018-10-31

**Authors:** Rajendra Chaudhary, Krishna Bahadur Raut, Kristina Pradhan

**Affiliations:** 1 Department of Obstetrics and Gynecology, Pokhara Academy of Health Sciences, Kaski, Nepal; 2 Department of Emergency, Chitwan Medical College, Chitwan, Nepal; 3 Kathamndu Medical College, Sinamangal, Kathmandu, Nepal

**Keywords:** *cesarean section*, *community hospital*, *indications*, *prevalence*

## Abstract

**Introduction:**

Cesarean section is one of the common obstetric procedures done when the childbirth is not anticipated to occur by the normal vaginal delivery. There has been increased rate of cesarean section globally as well as in our country in recent decades.

**Methods:**

This descriptive cross-sectional study has been carried out by reviewing a year of data from maternity ward of Paschimanchal Community Hospital, Prithvi Chowk, Pokhara. The total number of delivery, their modes either vaginal or cesarean, indications for the cesarean section and their outcomes were analyzed. The obtained data was entered and analyzed in Microsoft Excel.

**Results:**

Total of 257 cases underwent delivery during the study period and 174 (63.27%) were by cesarean section. Oligohydramnios is the most common indication for cesarean section. Around 25 (14.36%) of the women underwent repeat cesarean section.

**Conclusions:**

The rate of cesarean section was quite high in our study and further studies are recommended for understanding of causes and other associated factors with it.

## INTRODUCTION

Cesarean Section is one of the commonly done operative obstetric procedures when done with appropriate indication is a lifesaving procedure and helps in significant reduction of maternal mortality.^1^

According to the consensus made by WHO and other healthcare community on 1985, the ideal rate of cesarean section is supposed to be 10–15%.^2^ There has been worldwide increase in the trend of cesarean section to unprecedented levels in recent decades.^3^ In Nepal also, the rate of cesarean section has increased in recent years and is variable in different hospitals starting from 15% up to 81%.^4^ Women undergoing cesarean section in first pregnancy are more likely to deliver by cesarean section too in subsequent pregnancies and are in the risk of different operative and pregnancy related complications. ^5^

This study has been undertaken with an objective to find out the different indications of cesarean section and its outcome in a Community Hospital in Western Region of Nepal.

## METHODS

This descriptive cross-sectional study was carried out in the Paschimanchal Community Hospital, Prithivi Chowk, Pokhara, Kaski, a community based hospital with low cost for cesarean section and normal vaginal delivery among the other private hospitals in western region of Nepal. The hospital records of the patients who underwent normal delivery and cesarean section during the period of Baisakh 2074 to Chaitra 2074 was taken from hospital records section. Number of patients who have undergone normal vaginal delivery, cesarean section and their outcomes were reviewed. Convenience sampling was done to select the sample size. Ethical approval was taken from the respective authority. The obtained data was entered in Microsoft Excel and the descriptive statistical analysis was done.

## RESULTS

**Figure 1.**
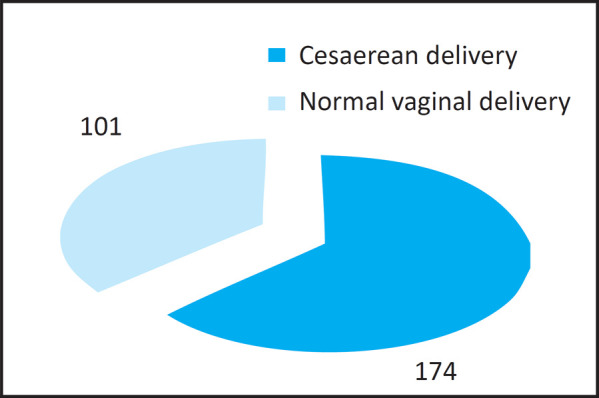
Percentage of vaginal and cesarean delivery.

Total of 275 cases underwent the delivery during the study period. Among them 174 (63.27%) were done via cesarean delivery while remaining 101 (36.72%) were done via normal vaginal delivery (Figure 1).

**Figure 2.**
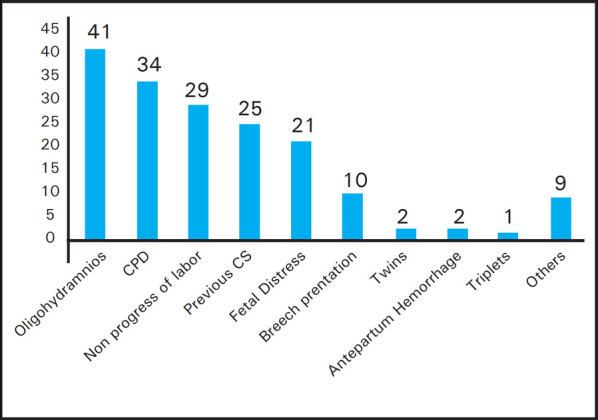
Different indications of cesarean section during the study period._

The common indications of cesarean section were oligohydramnios, cephalopelvic disproportion, non progress of labor, previous CS, fetal distress and so on. Oligohydramnios accounts for 41 (23.56%) and hence the most common indication (Figure 2).

**Figure 3.**
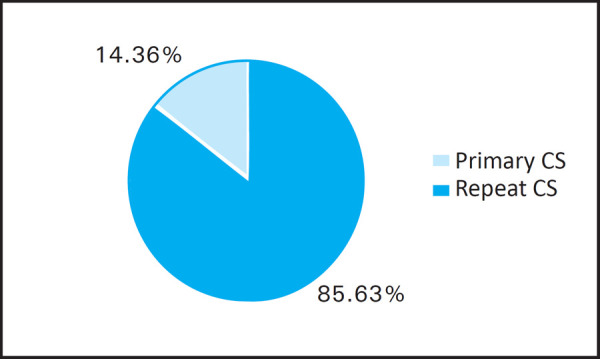
Primary and repeat CS during the study period.

Similarly, among the total cases, 149 (85.63%) of the cases are primary cesarean section and 25 (14.36%) are repeat cesarean section (Figure 3).

There was no any maternal mortality during the study period but two cases of vaginal delivery were intrauterine fetal deaths.

## DISCUSSION

Cesarean section has been boon for the mothers and newborn child in sense of preventing life threatening complications of pregnancy since nineteenth century.^6^ The increasing trend of cesarean among the pregnant women have negative future consequences and has the risk of increased maternal and pregnancy related complications in the subsequent days to come.^7, 8^ Following the international trends, there has been increase of cesarean section in Nepal too in last few decades.^9–11^ The rate of cesarean section is variable and ranges from 15% to 81% in various government and private hospitals of Nepal.^4^

Our study shows the rate of cesarean section among the total delivery as 63%. This rate is quite high among the similar study conducted in India as well as other hospitals of Nepal.^10–2^ The cause of high rates of cesarean in our center may be that Pokhara being the city with major hospitals in western region, the complicated cases from peripheral health institutions requiring cesarean section are referred here. Also due to the lack of beds and large number of patients in government hospital of Pokhara, those requiring the immediate surgery for delivery are referred to this hospital.

The most common indications for cesarean section in our study is oligohydramnios-23.56%, CPD-19.54%, non-progress of labor-16.67%, fetal distress-12.06% and so on in the decreasing order. Interestingly, other studies in similar settings in Nepal have meconium stained liquor, previous CS and fetal distress as the common indications. ^9–11^

Also, in our study among the total, around 14.36% of the cesarean section are repeated ones. This is also quite higher than the results in other similar studies. ^10, 11^

There has been controversy and debate regarding the usefulness and high increasing trend of cesarean section in recent years. A study by Barber et al. has suggested that the subjective indications like: arrests of dilation of cervix, non-reassuring fetal status are the causes for increasing primary cesarean section.^13^ However, serial sonographic monitoring for oligogydramnios, para cervical amino infusion for fetal distress, monitoring labor through partograph can be dome for reduction of primary cesarean section and promoting normal delivery. ^14^ Defensive obstetric practice and cesarean delivery on maternal request are also a significant factor not to be forgotten for the increase in its rate.^15, 16^

As this study was conducted in one hospital in Pokhara, so these results cannot be generalized for the whole country and the city, Pokhara. As this study uses convenience sampling, there could have been selection bias in selection of participants as participants with complications are usually referred from other hospitals to this community hospital. The main source of data in the study is from the hospitals records so there can be reporting bias in this study and some extent of error can also be present while entering the data to the analyzing software. To minimize such bias in the study, we have reviewed our results with results of previous studies and large sample size has also been taken. Rechecking was also done after entering the data. Furthermore studies are recommended in our settings to explore much regarding indications and the cause of increasing cesarean sections.

## CONCLUSIONS

The rate of cesarean section is found to be quite higher in our study than studies conducted in similar settings. Most of the cases underwent cesarean sections and on further analysis, we found oligohydraminos to be the most common indication of the cesarean section.

## Conflict of Interest

**None.**
